# The multifaceted role of ferroptosis in liver disease

**DOI:** 10.1038/s41418-022-00941-0

**Published:** 2022-01-24

**Authors:** Junyi Chen, Xiaopeng Li, Chaodong Ge, Junxia Min, Fudi Wang

**Affiliations:** 1 The First Affiliated Hospital, The Fourth Affiliated Hospital, Institute of Translational Medicine, School of Public Health, Cancer Center, State Key Laboratory of Experimental Hematology, Zhejiang University School of Medicine, 310058 Hangzhou, China; 2 The First Affiliated Hospital, The Second Affiliated Hospital, Basic Medical Sciences, School of Public Health, Hengyang Medical School, University of South China, 421001 Hengyang, China

**Keywords:** Translational research, Autophagy, Experimental models of disease

## Abstract

Ferroptosis is an iron-dependent form of non-apoptotic cell death characterized by excessive lipid peroxidation and associated with a plethora of pathological conditions in the liver. Emerging evidence supports the notion that dysregulated metabolic pathways and impaired iron homeostasis play a role in the progression of liver disease via ferroptosis. Although the molecular mechanisms by which ferroptosis causes disease are poorly understood, several ferroptosis-associated genes and pathways have been implicated in liver disease. Here, we review the physiological role of the liver in processing nutrients, our current understanding of iron metabolism, the characteristics of ferroptosis, and the mechanisms that regulate ferroptosis. In addition, we summarize the role of ferroptosis in the pathogenesis of liver disease, including liver injury, non-alcoholic steatohepatitis, liver fibrosis, liver cirrhosis, and hepatocellular carcinoma. Finally, we discuss the therapeutic potential of targeting ferroptosis for managing liver disease.

## Facts

- Liver disease is on the rise and now accounts for approximately two million deaths annually worldwide.
- Several forms of programmed cell death, including apoptosis, necroptosis, and ferroptosis, have been implicated in the pathogenesis of various liver diseases. With respect to liver injury, steatohepatitis, fibrosis, and cirrhosis, liver cells are more susceptible to ferroptosis; in contrast, in liver cancer the cancer cells are either intrinsically resistant to ferroptosis or acquire resistance to ferroptosis.
- A growing body of evidence suggests that ferroptosis may serve as a promising target for the prevention and treatment of many forms of liver disease.

## Open questions

- What physiological role, if any, does ferroptosis play in the liver?
- Can we identify reliable, sensitive biomarkers of ferroptosis in liver disease?
- When should we target ferroptosis in specific pathological liver conditions and/or disease stages?
- In treating liver cancer, can we activate ferroptosis specifically in cancer cells without affecting healthy cells?

## Introduction

Liver disease is a major cause of death worldwide [1], and cell death plays a key role in driving the progression of various forms of liver disease. With respect to regulated cell death, emerging evidence indicates that both necroptosis and ferroptosis play important roles in the pathogenesis of liver disease [2–5]. The pathophysiological relevance of ferroptosis was first demonstrated in vivo in ischemia/reperfusion injury (IRI) both in the liver and the kidneys [4]; since then, numerous studies have shown that lipid peroxidation and ferroptosis play a central role in a plethora of liver disease models. Moreover, blocking hepatic cell death can provide a cost-effective strategy for protecting the liver against injury and related diseases.

Ferroptosis is an iron-dependent form of cell death accompanied by large levels of lipid peroxidation and is distinct from other types of cell death such as apoptosis, autophagy, and pyroptosis [6, 7]. In addition to the role of iron metabolism in ferroptosis, an increasing number of other metabolic pathways have been implicated in ferroptosis, including the cyst(e)ine/glutathione (GSH)/glutathione peroxidase 4 (GPX4) axis, the guanosine triphosphate cyclohydrolase 1 (GCH1)/tetrahydrobiopterin (BH_4_)/dihydrofolate reductase (DHFR) axis, and the ferroptosis suppressor protein 1 (FSP1)/ coenzyme Q (CoQ) axis [8]. A growing number of studies suggest that ferroptosis plays an important role in the pathogenesis of various types of liver disease, including hemochromatosis, alcohol-associated liver disease (ALD), hepatitis C virus (HCV) infection, non-alcoholic steatohepatitis (NASH), and hepatocellular carcinoma (HCC) [9, 10]. Thus, targeting ferroptosis may provide a promising new therapeutic strategy for treating patients with liver disease.

Here, we review current knowledge regarding the role of ferroptosis in various forms of liver disease, and we provide new perspectives regarding the physiological role of the liver in processing nutrients and iron metabolism, as well as the potential mechanisms that underlie hepatic cell death and the pathophysiological pathways involved in liver disease.

### Hepatic metabolism of glucose, lipids, amino acids, and ferroptosis

**Fig. 1:**
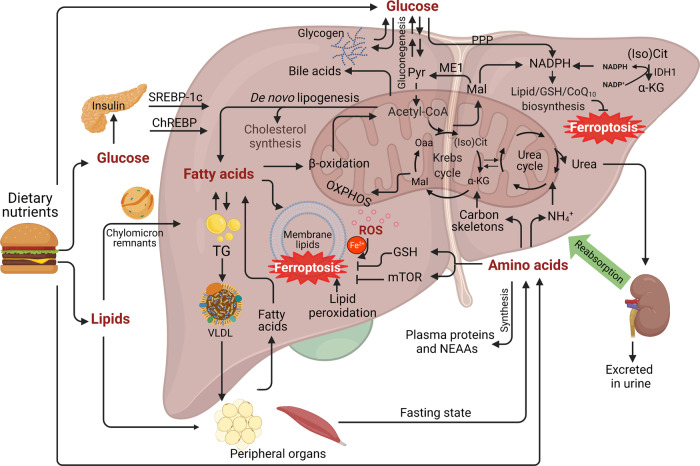
Hepatic glucose, lipids, and amino acid metabolism. The liver is an essential organ for controlling many physiological metabolic processes, including the metabolism of glucose, lipids, and amino acids. After a meal, glucose rapidly enters the liver and is sequestered as glycogen. Glucose produces energy via glycolysis and generates NADPH the PPP pathway. Two other major pathways also participate in the generation of NADPH by ME1 and IDH1. In addition, excess glucose stimulates de novo lipogenesis in the liver. During fasting, the liver secretes glucose both by breaking down glycogen (glycogenolysis) and by de novo glucose synthesis (gluconeogenesis). With respect to lipid metabolism, glycolytic products are used to synthesize fatty acids through de novo lipogenesis. The majority of chylomicrons are delivered to the adipose tissue and skeletal muscle, while chylomicron remnants are cleared by the liver. VLDL cholesterol is also assembled in the liver from triglycerides, cholesterol, and apolipoproteins, and used to transport endogenous TG to other peripheral organs. The liver is also the site of deamination, amino acid transamination, and urea synthesis. SLC7A11-mediated cystine uptake is essential for GSH synthesis, which inhibits ferroptosis. As a sensor of amino acids, mTORC1 activation can suppress ferroptosis by increasing GPX4 levels. Finally, altered glucose, lipid, and amino acid metabolism can lead to insulin resistance, NAFLD, and cholestasis. α-KG, α-ketoglutarate; ChREBP, carbohydrate-responsive element-binding protein; GSH, glutathione; IDH1, isocitrate dehydrogenase 1; Mal, malate; (Iso) Cit, (Iso)citrate; ME1, malic enzyme 1; mTOR, mechanistic target of rapamycin; NADPH, nicotinamide adenine dinucleotide phosphate; NAFLD, non-alcoholic fatty liver disease; NEAAs, non-essential amino acids; Oaa, oxaloacetate; OXPHOS, oxidative phosphorylation; PPP, pentose phosphate pathway; Pyr, pyruvate; ROS, reactive oxygen species; SREBP1c, sterol regulatory element-binding transcription factor 1c; TG, triglyceride; VLDL, very low-density lipoprotein.

The liver functions as the body’s metabolic hub for nutrients such as glucose, lipids, and amino acids (Fig. 1). Dysregulation of these macronutrients can lead to oxidative stress, liver disease, and even mortality [11, 12]. An increasing number of hepatic metabolic pathways have been associated with ferroptosis [13, 14]. For instance, NADPH levels decrease significantly upon induction of ferroptosis [14]. The metabolic NADPH pathway protects against ferroptosis by mediating the synthesis of GSH and CoQ_10_; thus, NADPH has been used as a biomarker for ferroptosis [15–18].

The metabolism of essential fatty acids (EFAs) is particularly important, as the accumulation of lipid peroxidation is considered one of the major characteristics of ferroptosis [19]. Non-alcoholic fatty liver disease (NAFLD) is characterized by abnormal lipid deposition in the liver. Importantly, prolonged overnutrition can increase the progression of NAFLD to NASH with fibrosis [20, 21].

Some amino acids are linked directly to ferroptosis by regulating oxidative stress. For example, cysteine is the limiting amino acid in GSH synthesis, and inhibiting its import through the cystine/glutamate antiporter SLC7A11 is sufficient to induce ferroptosis by depleting GSH levels [22]. Importantly, cystine deprivation has been shown to induce ferroptosis in a wide range of cancer cell lines. However, Conlon et al. [23] recently reported that cystine deprivation failed to induce ferroptosis when other amino acids such as arginine are also absent, suggesting that amino acids play a complex role in mediating the cellular response to ferroptosis inhibitors.

### Both systemic and hepatic iron hemostasis are tightly regulated

**Fig. 2:**
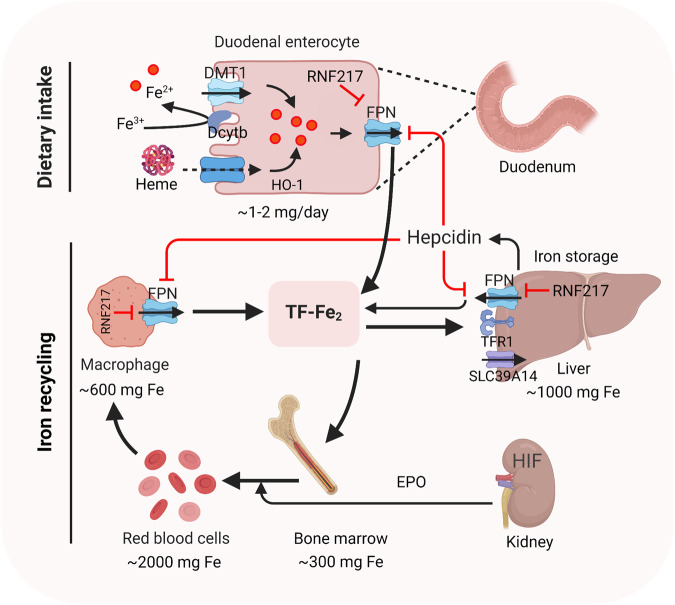
Regulation of systemic iron homeostasis. After intake of iron, Fe^3+^ is reduced by dcytb and then transported into enterocyte through DMT1. Dietary heme is absorpbed by unknown mechanism and degraded in enterocyte by HO-1. Once exported by FPN, Fe^3+^ binds to transferrin (diferric transferrin, TF-Fe_2_), travels to tissues, and largely utilized in new red blood cells. Macrophage degraded senescent RBCs to recycle iron. Once needed, EPO, released by kidney, promotes erythropoiesis by HIF signaling pathway. The iron utilization of erythroid marrow and its recycling by macrophages represent the major iron circulation. Excess iron can be stored in hepatocytes through TFR1-mediated TF-Fe_2_ or SLC39A14-participated non-transferrin-bound iron (NTBI). The release of iron from enterocyte, red blood cells, and macrophages is precisely controlled by FPN, the body’s sole iron exporter, to maintain a relatively stable iron level. The peptide hepcidin, the master regulator of systemic iron homeostasis, is a circulating hormone synthesized by the liver. Recently, we identified RNF217 as a novel E3 ligase for mediating FPN degradation. Dcytb, duodenal cytochrome b; DMT1, divalent metal transporter 1; EPO, erythropoietin; FPN, ferroportin; TFR1, transferrin receptor 1; HO-1, heme oxygenase 1; HIF, hypoxia induced factor; RBCs, red blood cells; NTBI, non-transferrin-bound iron.

**Fig. 3:**
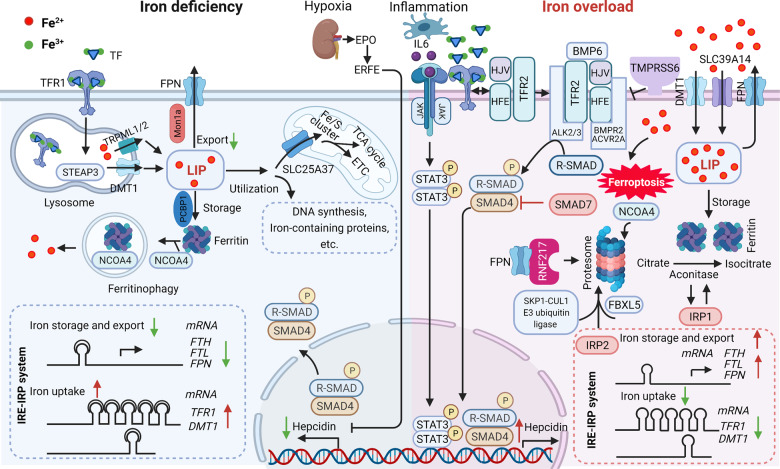
Iron metabolism in the liver. Under iron-deficient conditions (left), the majority of iron is bound to transferrin (TF), which binds to the transferrin receptor 1 (TFR1) at the cell surface followed by receptor-mediated endocytosis, resulting in ferric iron being released from TF and reduction to ferrous iron by an lysosomal reductase such as STEAP3. The ferrous iron is then transported into the lysosomal membrane by DMT1 and TRPML1/2, where it becomes part of the labile iron pool in the cytosol. Labile iron can be stored in the iron-storage protein ferritin or used to synthesize heme and iron-sulfur clusters in the mitochondria or in the cytosol. Iron can also be exported from the cell by the body’s sole iron exporter, ferroportin (FPN). In addition, the IRE/IRP system regulates the expression of iron-related proteins such as TFR1, ferritin and FPN, upregulating TFR1 and DMT1 expression and downregulating FPN and FTH/FTL expression. During iron overload (right), hepcidin expression is upregulated by either the canonical bone morphogenetic protein (BMP)/SMAD pathway or by IL-6-pSTAT3 inflammatory signaling, which in turn limits iron absorption by increasing FPN degradation. In response to excess iron, BMP6, together with HJV, activates type 1 (Alk2/3)and type 2 (BMPR2, ACVR2A) BMP serine threonine kinase receptors to phosphorylate R-SMAD (receptor-activated SMAD), leading to activation of BMP/SMAD signaling pathway. High concentration of TF-Fe_2_ interact with TFR1, resulting in forming complex of TFR2/HJV/HFE to enhance the BMP/SMAD signaling in regulating hepcidin. TMPRSS6 inhibits BMP/SMAD signaling by cleaving HJV. The IRP system not only downregulates iron uptake-related genes such as TFR1 and DMT1 expression, it also upregulates FPN and FTH/FTL expression. IRP2 mediated by SKP1-CUL1 E3 ubiquitin ligase and NCOA4 are degraded, while IPR1 works as aconitase to convert citrate to isocitrate due to conformational change. RNF217 is a recently identified E3 ligase that regulates the degradation of FPN. Non-transferrin-bound iron (NTBI) enters hepatocytes through metal transporter proteins such as SLC39A14, and the increased iron can directly induce ferroptosis. ACVR2A, activin receptor type-2A; ALK, activin receptor-like kinase; BMP6, bone morphogenetic protein 6; BMPR2, bone morphogenetic protein receptor type 2; DMT1, divalent metal transporter 1; EPO, erythropoietin; ERFE, erythroferrone; ETC, electron transport chain; FBXL5, F-box/LRR-repeat protein 5; FPN, ferroportin; FTH, ferritin heavy chain; FTL, ferritin light chain; JAK, Janus kinase; LIP, labile iron pool; NCOA4, nuclear receptor coactivator 4; NTBI, non-transferrin-bound iron; HJV, hemojuvelin; IL-6, interleukin 6; IRE, iron-responsive elements; IRP, iron-regulatory proteins; SLC39A14, solute carrier family 39 member 14; SMAD4, SMAD family member 4; SMAD7, SMAD family member 7; STAT3, signal transducer and activator of transcription 3; STEAP3, six-transmembrane epithelial antigen of prostate 3; TCA cycle, tricarboxylic acid cycle; TFR1, transferrin receptor 1; TFR2, transferrin receptor 2; TMPRSS6, transmembrane protease serine 6; TRPML1/2, Mucolipin TRP channel 1/2;UTRs, untranslated regions.

Systemic iron homeostasis is tightly regulated in order to ensure that the body contains sufficient iron due to dietary iron absorption and to prevent the toxic accumulation of iron (Fig. 2). In addition, iron metabolism is also regulated primarily by the liver, which maintains systemic iron balance by producing and secreting hepcidin, the master regulator of iron homeostasis [24] (Fig. 2). Hepatocytes acquire transferrin-bound iron (TBI) and non-transferrin-bound iron (NTBI) via TFR1 and SLC39A14, respectively [25–27]. In hepatocytes, iron is stored in ferritin and exported by ferroportin (FPN) [28] (Fig. 3). Mon1a was identified as a modifier gene of cellular iron metabolism via trafficking of FPN to the surface of iron-recycling macrophages [29]. Hepcidin inhibits cellular iron efflux by binding to and inducing the degradation of FPN. RNF217 was identified as a novel E3 ubiquitin ligase and was shown to play a role in mediating the degradation of FPN [30]. Notably, we previously showed that iron overload can directly induce ferroptosis in a variety of organs, including the liver and heart [14, 26, 31].

### Dysregulated iron metabolism triggers ferroptosis

Lipid peroxidation and toxic ROS result from the iron-mediated Fenton reaction and enzymatic oxygenation, which are hallmark features of ferroptosis [32]. Impaired iron homeostasis plays a role in the pathogenesis of various diseases by triggering ferroptosis, with recent reports implicating transferrin and its cell surface TFR1 as key regulators of ferroptosis [33]. Interestingly, CISD1 (CDGSH iron-sulfur domain‒containing protein 1, also known as MitoNEET) inhibits ferroptosis by altering the accumulation of mitochondrial iron [34]. We recently showed that hepatic SLC39A14 accelerates iron-induced ferroptosis in the liver of hepatic *Trf* knockout mice via its ability to transport NTBI, revealing a novel molecular mechanism by which ferroptosis drives the pathogenesis of liver fibrosis and cirrhosis [26].

**Fig. 4:**
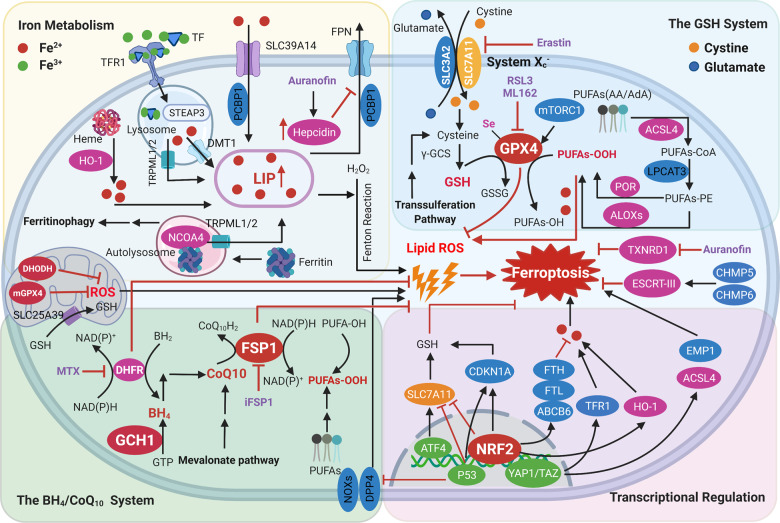
Regulatory signaling pathways and defense mechanisms implicated in the process of ferroptosis. Iron metabolism has been associated with ferroptosis (top left). As the body’s principal organ for storing excess iron and synthesizing hepcidin, the liver serves as the functional cornerstone in maintaining iron homeostasis. When cellular iron is sufficient, transferrin-bound iron decreases in order to limit excessive iron accumulation. Under iron-overload conditions, excess iron results in redox-active non-transferrin-bound iron (NTBI), the uptake of which is mediated by metal transporter proteins such as SLC39A14. In general, excess iron is stored in ferritin and free iron existing as part of the LIP can be accumulated due to imbalanced metabolism. NOCA4 has been shown to act as a cargo receptor that binds to ferritin delivering it to the autolysosome, resulting in releasing of free iron. Free iron participates in the generation of ROS through Fenton reaction. ACSL4 and LPCAT3 are necessary for ferroptosis to produce PUFAs-PE. The activation of ALOXs, POR, or NOXs promotes lipid peroxidation, yet the activation of ESCRT complex repairs cell membrane damage. Lipid peroxidation of membrane phospholipids can be eliminated by three parallel metabolic pathways, including the cyst(e)ine/GSH/GPX4 axis (top right), as well as the CoQ_10_/FSP1 and GCH1/BH4/DHFR axis (bottom left). In mitochondrial, DHODH and mitoGPX4 participated in ROS scavenging. In addition, several transcription factors such as p53, NRF2, ATF4, and YAP/TAZ regulate ferroptosis-related genes upon oxidative stress (bottom right). ABCB6, ATP-binding cassette subfamily B member 6; LOXs, lipoxygenases; ACSL4, acyl-CoA synthetase long-chain family member 4; ATF4, activating transcription factor 4; BACH1, BTB domain and CNC homolog 1; BH_2_, 7,8-dihydrobiopterin; BH_4_, tetrahydrobiopterin; CDKN1A, cyclin-dependent kinase inhibitor p21; CHMP5/6, chromatin modeling protein 5/6; CoQ_10_H_2_, ubiquinol; GCH1, guanosine triphosphate cyclohydrolase 1; DHFR, dihydrofolate reductase; DHODH, dihydroorotate dehydrogenase; DDP4, dipeptidyl peptidase-4; DMT1, divalent metal transporter1; EMP1, epithelial membrane protein 1; ESCRT-III, endosomal sorting complex required for transport III; FPN, Ferroportin; FSP1, ferroptosis suppressor protein 1; FTH, ferritin heavy chain; FTL, ferritin light chain; GCS, glutamylcysteine synthetase; GPX4, glutathione peroxidase 4; GCH1, guanosine triphosphate cyclohydrolase 1; GSH, glutathione; HO-1, heme oxygenase 1; LIP, labile iron pool; LPCAT3, lysophosphatidylcholine acyltransferase 3; MTX, methotrexate; mTORC1, mechanistic target of rapamycin complex 1; NRF2, nuclear factor erythroid 2-related factor 2; NCOA4, nuclear receptor coactivator 4; PCBP, poly (RC)-binding proteins; POR, NADPH-cytochrome P450 reductase; PUFA, polyunsaturated fatty acid; ROS, reactive oxygen species; STEAP3, six-transmembrane epithelial antigen of prostate 3; TF, transferrin; TFR1, transferrin receptor 1; TXNRD1, thioredoxin reductase 1; TRPML1/2, Mucolipin TRP channel 1/2.

Increased iron uptake and reduced iron storage may lead to iron overload, subsequently triggering ferroptosis. This hypothesis was tested using iron chelators and iron supplements during erastin-mediated ferroptosis [17]. Nuclear receptor coactivator 4 (NCOA4)-mediated ferritinophagy, a specific form of autophagy, has been shown to induce ferroptosis by degrading ferritin and inducing iron overload [35]. The iron chaperone activity of PCBP1 is required for preventing iron-mediated toxicity and preventing ferroptosis [36]. Taken together, these studies indicate that iron metabolism is tightly regulated at multiple levels through the ferroptosis pathway (Fig. 4).

### Accumulation of lipid peroxides drives ferroptosis

Polyunsaturated fatty acids (PUFAs) containing *bis*-allylic carbons are highly susceptible to lipid peroxidation [37], and recent findings suggest that a wide range of PUFAs may be involved in the ferroptosis pathway [15]. Inhibiting related enzymes such as fatty acid desaturase 2 (FADS2) and acetyl-CoA carboxylase (ACC) confers resistance to lipid peroxidation and ferroptosis induced by the compound RSL3 [38, 39]. Recently, a genetic screen revealed two lipid metabolism regulators—lysophosphatidylcholine acyltransferase 3 (LPCAT3) and acyl-CoA synthetase long-chain family member 4 (ACSL4)—that drive ferroptosis via an accumulation of oxidized phospholipids in the cell membrane [40]. ACSL4 preferentially catalyzes the esterification of PUFAs, including AA, with CoA. PUFA-CoAs are subsequently used for the synthesis of phospholipids, including phosphatidylethanolamine, and loss of ACSL4 results in significant resistance to ferroptosis induced by GPX4 deficiency [41]; in contrast, knocking out LPCAT3 does not confer robust protection against ferroptosis, suggesting that these family members may have compensatory mechanisms [41]. It is important to note that “lipid peroxidation” is an ambiguous concept, as its products can form spontaneously by either autoxidation (mediated by carbon- and oxygen-centered radicals) or enzyme-catalyzed processes [42, 43]. In addition to the non-specific propagation of radicals, lipid peroxidation can also be accelerated by cyclooxygenases (COXs) [44], cytochrome p450 oxidoreductase (POR), and lipoxygenases (LOXs) via a controlled process [42, 45].

Whether a cell undergoes ferroptosis is largely determined by intertwined signals from subcellular organelles such as mitochondria, lysosomes, and peroxisomes, which coordinately regulate ROS production as well as lipid oxidation in the process of ferroptosis [46]. Emerging evidence supports the notion that mitochondria are an important regulator of ferroptosis through the integrative regulation of ROS production, the TCA cycle, iron metabolism, and mitochondrial DNA [47]. Lysosomes participate in ferroptosis primarily through the activation of selective autophagy processes (e.g., ferritinophagy and lipophagy) and by lysosomal accumulation of iron and nitric oxide [35, 48]. Using CRISPR screening and lipidomics profiling, Zou et al. [45] recently found that peroxisomes induce ferroptosis by synthesizing polyunsaturated ether phospholipids (PUFA-ePLs), which are substrates of lipid peroxidation. Although significant advances have been made toward unraveling the signaling pathways involved in the production of lipid peroxidation, the specific contribution of various organelles in modulating ferroptosis remains an open question.

### The GSH/GPX4 axis inhibits ferroptosis

The GSH/GPX4 axis plays an essential role in counteracting the production of specific phospholipid hydroperoxides in the presence of catalytically active iron. The upstream component in this system is system x_c_^-^, a cystine–glutamate antiporter composed of the transporter protein SLC7A11 linked via a disulfide bond to the regulatory subunit SLC3A2. Inhibition of system x_c_^-^ activates cellular ferroptosis in cell lines due to a lack of intracellular cysteine. The transsulfuration pathway, which transfers sulfur from homocysteine to cysteine, regulates ferroptosis in cells in which system x_c_^-^ is inhibited [49]. Recent studies found that transcription factors such as p53, the tumor suppressor BAP1 (BRCA1-associated protein 1), and ATF3 (activating transcription factor 3) increase cellular susceptibility to ferroptosis via decreased cystine uptake by inhibiting *SLC7A11* expression [50–52]. Interestingly, OTUB1 (OTU domain-containing ubiquitin aldehyde-binding protein 1) has also been shown to play a key role in SLC7A11 stability and sensitivity to ferroptosis [53].

GSH is generated by de novo synthesis in a process mediated by two ATP-dependent ligases (glutamate-cysteine ligase and glutathione synthase), as well as by the regeneration of GSH from GSSG, a process catalyzed by glutathione disulfide reductase. Endogenous cysteine is generated primarily via GSH or the TXNRD1 (thioredoxin reductase 1)-mediated reduction of cystine [54]. High doses of the anti-rheumatic drug auranofin have been shown to activate ferroptosis by inhibiting TXNRD1 activity [55].

Finally, GPX4 is a well-characterized core suppressor of ferroptosis that directly reduces lipid hydroperoxide to form non-toxic lipid alcohol. GSH serves as a cofactor for the selenoenzyme GPX4, and inhibiting the de novo synthesis of GSH synthesis induces ferroptosis by inactivating GPX4 [56]. GPX4 also plays a role in the development and maintenance of a variety of physiological functions, as global knockout of *Gpx4* in mice causes embryonic lethality, whereas conditional *Gpx4* knockout mice develop disorders in the endothelium, liver, brain, kidney, immune system, and hematopoietic system [57]. As a selenocysteine-containing enzyme, GPX4 has mitochondrial, cytosolic, and nuclear isoforms, all of which are encoded by the same gene. Mitochondrial or nuclear *Gpx4* deletion in mice causes male infertility without impairing embryogenesis or postnatal development [58, 59]. Together, these in vivo animal studies suggest that mitochondrial and/or nuclear GPX4 plays an important role in mammalian male fertility, while cytosolic GPX4 is essential for embryonic development and cell survival.

### The NAD(P)H/FSP1/CoQ_10_ system suppresses ferroptosis

To identify GPX4-independent ferroptosis-resistance genes, two groups independently performed a synthetic lethal CRISPR-Cas9 screen [15] and a gain-of-function screen [16]. Interestingly, both studies identified *FSP1* (encoding ferroptosis suppressor protein 1) as a novel ferroptosis-resistance gene [15, 16]. FSP1’s anti-ferroptotic function is independent of cellular GSH levels, GPX4 activity, ACSL4 expression, and oxidizable FA content, suggesting a new anti-ferroptosis mechanism [15, 16]. Overexpressing FSP1 in cells lacking *GPX4* expression was shown to significantly reduce specific phospholipid peroxidation products compared to control cells [15, 16]. Moreover, cells lacking *FSP1* expression have increased sensitivity to ferroptosis inducers, including the GPX4 inhibitor ML162 and the system x_c_^-^ inhibitor erastin [15, 16]. Recently, Dai et al. [51] found that FSP1 prevents ferroptosis by promoting ESCRT-III (endosomal sorting complex required for transport III)‒mediated membrane repair in a CoQ_10_-independent manner. Based on currently available evidence, both FSP1-CoQ_10_ and ESCRT-III play a role in repairing membrane damage during ferroptosis. Given that CoQ_10_ is an essential cofactor in the mitochondrial electron transport pathway, as well as a lipid-soluble antioxidant, it is reasonable to speculate that FSP1-CoQ_10_ and ESCRT-III have complementary functions. Taken together, these findings indicate that FSP1 blocks ferroptosis via a complex interplay between regulatory pathways.

### The GCH1/BH_4_/DHFR system suppresses ferroptosis

The metabolic change that occurs in cancer cells often requires endogenous antioxidants to reduce toxic lipid oxidation. Both the cyst(e)ine/GSH/GPX4 system and the NAD(P)H/FSP1/CoQ_10_ system have been implicated in the metabolic processes related to the cellular response to lipid peroxidation [15–17] (Fig. 4). Genome-wide CRISPR activation screening identified guanosine triphosphate cyclohydrolase 1 (GCH1), the rate-limiting enzyme in the synthesis of BH_4_, as a potent GPX4-independent ferroptosis-inhibiting factor. [60, 61]. The BH_4_ molecule serves as an essential cofactor for a number of enzymes involved in central metabolic processes, including tryptophan hydroxylases, nitric oxide synthase (NOS), alkylglycerol monooxygenase, and isoenzymes. Importantly, BH_4_ is used not only as a direct antioxidant to protect cells from lipid peroxidation and ferroptosis, but also in the synthesis of CoQ_10_ [60, 61]. Nevertheless, further studies are needed in order to elucidate the antioxidant mechanisms involving the GCH1/BH_4_/DHFR system in the context of ferroptosis.

### Mitochondrial GPX4 and DHODH suppress ferroptosis

Mitochondria are essential for maintaining cellular viability, as they perform critical functions in bioenergetics, metabolism, and various signaling pathways. The primary function of mitochondria is to generate ATP via oxidative phosphorylation using the electron transport chain. It has long been recognized that ROS are produced during mitochondrial respiration and that mitochondria serve as the cell’s principal source of ROS production. ROS trigger lipid peroxidation‒induced cell death by reacting with PUFAs in the lipid bilayers. Moreover, a number of molecular, cellular, pharmacological, and metabolomics studies indicate that mitochondrial metabolic activity is required for generating sufficient levels of ROS to trigger ferroptosis [47, 62]. In response to various forms of oxidative damage, cells use multiple protective and/or repair systems to reduce the effects of toxic modified membrane lipids. To prevent damage due to lipid peroxides, mammalian cells rely primarily on three systems involving GPX4, FSP1, and DHFR. Recently, however, Mao et al. [63] found that the enzyme dihydroorotate dehydrogenase (DHODH) and mitochondrial GPX4 constitute two additional defense enzymes that can detoxify lipid peroxides in the mitochondria. As an iron-containing flavin-dependent enzyme, DHODH couples the pyrimidine biosynthesis pathway to the mitochondrial respiratory chain, suggesting possible strategies for treating GPX4^low^ cancers by targeting DHODH [63].

### Oxidative stress-related transcription factors regulate ferroptosis

Ferroptosis is a highly complex and modifiable process that requires transcription factors to either repress or activate the expression of ferroptosis-related genes. The functions of various transcription factors such as TP53, NRF2, ATF4, YAP1, and TAZ in ferroptosis are summarized in Fig. 4. Among these transcription factors, NRF2 is a key negative regulator of ferroptosis [64]. The activity of NRF2 is mediated by the KEAP1/CUL3/RBX1/E3 ubiquitin ligase complex [65]. In contrast, under oxidative stress NRF2 dissociates from KEAP1 and translocates to the nucleus, where it activates the downstream expression of antioxidative response element (ARE)-containing target genes, many of which have been shown to play a role in defending against ferroptosis [65, 66]. Interestingly, activation of NRF2 regulates iron-related genes such as *FTH1*, *FTL1*, *SLC40A1*, *ABCB6*, and *HMOX1*, which in turn promotes the synthesis of GSH synthesis, limits ROS production, and regenerates NADPH [64, 66]. The protein p53 controls ferroptosis via two distinct mechanisms. On one hand, p53 has been shown to promote ferroptosis by repressing *SLC7A11* gene [52]. On the other hand, p53 has been shown to inhibit ferroptosis by negatively regulating dipeptidyl peptidase-4 (DDP-4) [67]. Depletion of ATF4 (activating transcription factor 4) has also been shown to increase erastin-induced ferroptosis and RSL3-induced ferroptosis in several cancer cell lines [68]. Finally, the Hippo pathway promotes ferroptosis by transcriptionally regulating the *TFRC* (which encodes TFR1) and *ACSL4* genes [69]. Thus, a wide range of transcription factors appear to coordinate cellular sensitivity to ferroptosis.

### Ferroptosis triggers acute liver injury

ALI is characterized by a rapid decline in hepatocyte function in patients without evidence of pre-existing liver disease [70]. In the majority of cases, ALI results from hepatotoxicity due to drugs, alcohol, ischemia/reperfusion injury (IRI), or viral hepatitis [70]. Among the long list of drugs that can cause hepatotoxicity, the canonical nonsteroidal anti-inflammatory drug acetaminophen (N-acetyl-para-aminophenol, or APAP) has probably been the most thoroughly documented. Lőrincz et al. [71] reported that ferroptosis occurs in APAP-induced hepatic injury, showing that ferroptosis inhibitors such as ferrostatin-1 (Fer-1) provided moderate protection against APAP-induced death in primary mouse hepatocytes. Recently, Yamada et al. [72] reported that ferroptosis occurs in a mouse model of APAP-induced acute liver failure, showing elevated lipid peroxides derived from *n*-6 PUFAs. In addition, both the ferroptosis inhibitor UAMC-3203 and the VDAC1 oligomerization inhibitor VBIT-12 have been shown to reduce ferroptosis in APAP-induced ALI animal model by protecting mitochondrial function [73]. Taken together, these studies suggest that ferroptosis may serve as a therapeutic target for APAP-induced ALI. Nevertheless, additional data are needed, as other forms of cell death might compensate if one form—for example, ferroptosis—is targeted.

Hepatic IRI-induced ALI can occur in a wide range of clinical contexts, including liver transplantation, systemic shock, heart failure, hemorrhage, and sepsis [74]. Many studies suggest that a complex network involving various types of cell death, including apoptosis and necroptosis, contributes to hepatic IRI. Notably, both liver IRI and kidney IRI are significantly reduced by the ferroptosis inhibitor liproxstatin-1 (Lip-1), suggesting that ferroptosis plays an important pathogenic role in IRI [4]. GPX4 has also been functionally characterized as a critical protector of both hepatic and renal function; importantly, α-tocopherol was also shown to protect against excessive lipid peroxidation in *Gpx4* knockout mice [75]. Recently, a study involving human subjects found that elevated serum ferritin levels in liver donors were associated with a significant higher risk of liver damage in transplantation recipients [76], and blocking ferroptosis with compounds such as Fer-1, α-tocopherol, and DFO prevented hepatic IRI [76]. Finally, the human liver is composed of several cell types, including hepatocytes, endothelial cells, Kupffer cells and other immune cell types, bile duct cells, and epithelial progenitor cells; however, the cellular composition and contribution of each cell type during ALI is poorly understood and warrants further study.

### Ferroptosis increases chronic liver injury and fibrosis

The majority of liver fibrosis cases were attributed to chronic hepatitis B virus (HBV) and/or hepatitis C virus (HCV) infection [77]. Both HBV and HCV infections alter hepcidin levels and a variety of other iron-related parameters that are closely linked to liver fibrosis [78]. A subset of immune cells, including macrophages, neutrophils, T cells, and B cells, have been implicated in ferroptosis or are intrinsically sensitive to ferroptosis due to changes in gene expression during maturation, which can significantly affect both innate and adaptive immunity, as recently reviewed in detail by Chen et al. [79].

Whether ferroptosis plays a role in the progression of HBV/HCV infection and infection-related liver diseases such as fibrosis remains an open question. Recently, Yamane et al. [80] reported that ferroptosis plays an important role in HCV replication. Interestingly, ferroptosis inhibitors reduce the antiviral activity of direct-acting antiviral medications (DAAs) against HCV [80]. In addition, the enzyme fatty acid desaturase 2 (FADS2) converts oleate to Mead acid and other highly unsaturated fatty acids, which act as a key regulator of HCV replication by promoting lipid peroxidation [80]. Altogether, these important findings may pave the way to developing novel antiviral strategies for treating infectious liver disease.

Excess iron has long been postulated as a risk factor for developing liver fibrosis and cirrhosis, and recently direct evidence has emerged with respect to the pathogenic role of ferroptosis in iron overload-induced liver damage and fibrosis [14]. However, the precise function of iron-regulating proteins in the development of liver fibrosis remains poorly understood. From this perspective, it is interesting to note that we recently reported that transferrin plays an important role in both high dietary iron-induced liver fibrosis and carbon tetrachloride (CCl_4_)-induced liver fibrosis in mice, and inhibiting ferroptosis potently prevented these effects; in addition, we found that clinical data support the role of transferrin in protecting against liver fibrosis by blocking ferroptosis, providing a possible therapeutic target for preventing ferroptosis-induced liver fibrosis [26].

The transdifferentiation of hepatic stellate cells (HSCs) into matrix-producing myofibroblasts is considered a key step in the development of liver fibrosis [81]. Recent studies demonstrate the potential of inducing ferroptosis in HSCs using magnesium isoglycyrrhizinate, artesunate, or artemether as a therapeutic strategy designed to mitigate the development of liver fibrosis [82, 83]. Moreover, certain regulators of ferroptosis in HSCs, including p53, ELAV-like protein 1 (ELAVL1), and zinc finger protein 36 (ZFP36), have been reported as promising targets in preventing liver fibrosis [84, 85]. Taken together, these studies indicate that inducing ferroptosis in HSCs may represent a viable strategy for treating and/or preventing liver fibrosis; however, a remaining challenge is the ability to selectively induce ferroptosis in HSCs with minimal effects on healthy hepatocytes.

### Ferroptosis and steatohepatitis

Steatosis and steatohepatitis are two pathological manifestations of liver disease. Ferroptosis was recently associated with the onset of inflammation in steatohepatitis in the early stages of NASH in mouse models[13]. Additional studies support the notion that ferroptosis and lipid metabolic disorders play an essential role in the progression of NASH, while—conversely—inhibiting ferroptosis significantly decreases the severity of NASH [86, 87]. Moreover, 12/15-lipoxygenase has been functionally connected to hydroperoxy-phosphatidylethanolamine-related ferroptosis [88]. Recently, arachidonate 12-lipoxygenase (ALOX-12) promotes the progression NASH via its interaction with acetyl-CoA carboxylase 1 (ACC1) [89, 90]. However, whether ferroptosis plays a role in ALOX-12‒mediated NASH remains an open question. Taken together, these results suggest that ferroptosis may serve as a potential therapeutic target in patients with NASH. Future studies are needed to identify the precise molecular mechanisms underlying ferroptosis and lipid metabolism in the pathogenesis of NASH.

### Targeting ferroptosis to treat HCC

Globally, HCC is the third leading cause of cancer-related death. Since the approval of sorafenib, the only first-line therapy for advanced HCC. Altered regulation of iron metabolism has been shown to play a role in the pathogenesis of HCC, particularly in patients who present with NASH, hemochromatosis, or thalassemia [91, 92]. High intake of dietary iron can also increase the risk of HCC, suggesting that high iron content may promote the development of HCC [93]. Moreover, a growing body of evidence supports the notion that activating ferroptosis may potently inhibit the growth of HCC cells, thus providing a scientific rationale for targeting ferroptosis as a novel therapeutic strategy for HCC.

Several studies using HCC cell lines suggests that ferroptosis may serve as the principal mechanism underlying the anticancer effect of sorafenib via suppressing the cystine/glutamate antiporter SLC7A11 [94–96]. Interestingly, retinoblastoma (Rb)-negative HCC cells are more susceptible to sorafenib-induced cell death compared to Rb-expressing HCC cells [97]. It should be noted that a recent study found that sorafenib was not a bona fide inducer of ferroptosis based on screening results using a panel of cancer cell lines [98]. Another recent study found that LIFR (leukemia inhibitory factor receptor) sensitized HCC cell lines to sorafenib-induced ferroptosis both in vitro and in vivo, whereas loss of *LIFR* expression confers resistance to sorafenib-induced ferroptosis [99]. These contradictory results might be attributed to different cellular contexts such as the expression levels of LIFR and/or other undefined genes/pathways related to the sensitivity of sorafenib.

Recently, IFNγ was used to sensitize HCC cells to ferroptosis by suppressing system x_c_^-^ via activation of the JAK/STAT signaling pathway, thus providing new insights into the feasibility of using IFNγ to induce ferroptosis in treating HCC [100]. In addition, the progression of HCC was recently linked to the circ0097009/miR-1261/SLC7A11 axis [101]. Interestingly, suppressing FTH renders HCC cells more sensitive to both RSL3- and iron-induced ferroptosis. Conversely, HCC cells with increased extracellular lactate levels are more resistant to ferroptosis induced by RSL3 and erastin [102]. Taken together, these studies suggest that inducing ferroptosis may serve as promising strategy for treating HCC.

### Therapeutic opportunities

#### Ferroptosis inducers

Many ferroptosis inducers and ferroptosis-related genes and pathways have been discovered [7] (Table 1). In addition to genetic regulators, intracellular iron accumulation, excessive lipid peroxidation, and small molecules can also trigger ferroptosis [6, 17]. Thus, ferroptosis-inducing compounds (FINs) are widely accepted as a promising approach for developing novel cancer agents. Based on their mode of action, FINs are categorized into four classes. Class I FINs activate ferroptosis by depleting intracellular GSH by inhibiting system X_c_^-^ [17, 95, 103, 104]. Class II FINs induce ferroptosis by directly inactivating GPX4, such as RSL3 and ML162, which covalently bind to GPX4 to suppress its function, leading to an accumulation of toxic lipid peroxides and inducing ferroptosis [17, 105]. Notably, persistent (i.e., drug-tolerant) cancer cells are particularly vulnerable to GPX4 inhibitors. Moreover, efforts are currently being made to develop GPX4-selective covalent inhibitors with minimal toxicity and better tolerance profiles [106, 107]. Class III FINs such as FIN56 act by indirect inhibiting and inactivating GPX4 via the squalene synthase-mevalonate pathway, which participates in the mitochondrial electron transport chain and also functions as endogenous lipophilic antioxidants [40, 108]. Finally, class IV FINs induce ferroptosis by causing iron overload or by activating HO-1 [109].

**Table 1:** Genetic and pharmacologic inducers of ferroptosis.

Target	Effector/reagent	Proposed mechanism	Reference(s)
Genetic activators			
SLC7A11	p53	Inhibits system X_c_^-^	[52]
SLC7A11	BAP1	Represses SLC7A11 expression	[50]
Iron transport	SLC39A14	Imports iron and increases intercellular iron levels	[26]
Transferrin	TFR1	Uptakes transferrin-iron	[33, 118]
Iron homeostasis -related mRNA	IRP2	Regulates iron level by regulating the translation and stability of mRNA	[17, 118]
Free fatty acids	ACSL4	Converts free fatty acids into fatty CoA ester	[4]
Polyunsaturated fatty acids	LOXs	Promotes peroxidation of polyunsaturated fatty acids	[111]
NADPH	NOXs	Induces lipid ROS generation	[17]
Polyunsaturated phospholipid	POR, CYB5R1	Enables membrane polyunsaturated phospholipid peroxidation	[45, 119]
Lysophospholipids	LPCAT3	Biosynthesis of phospholipids	[41]
Squalene	SQS	Synthesizes squalene and participant in cholesterol synthesis	[108, 120]
Squalene	SQLE	Converts squalene to squalene-2,3 epoxide and participtes in cholesterol synthesis	[120]
Ferritin	NCOA4	NCOA4-mediated ferritinophagy	[121]
Heme	HO-1	Degrades heme; releases free iron	[31]
Pharmacologic inducers			
Inhibition of system Xc-	Erastin, PE, IKE, other erastin analogs, sulfasalazine, sorafenib, glutamate	Blocks cysteine import, inducing GSH depletion and GPX4 inactivation	[17, 95, 97, 103, 104]
Inhibition of GPX4	RSL3, ML162, ML210, JKE1674, compound 26a	Covalently inhibits GPX4, causing accumulation of lipid peroxidation	[17, 105, 107, 122]
SQS and GPX4	FIN56, CIL56	Depletes CoQ_10_ via the SQS-mevalonate pathway and reduces GPX4 protein abundance	[40, 108]
Lipid peroxidation	FINO_2_	Oxidizes iron, inducing lipid peroxidation and indirectly inactivation of GPX4	[123]
HMG	Fluvastatin, lovastatin acid, simvastatin	Depletes of CoQ_10_	[108, 124, 125]
Iron	Ferric ammonium citrate	Increases iron abundance	[17]
GSH	BSO, DPI2, cisplatin, acetaminophen	Depletes GSH	[71, 103]
TXNRD1	Auranofin	Inhibits thioredoxin reductase	[55]
Ferroportin, transferrin	Siramesine, lapatinib	Increases iron abundance	[126]
NRF2	Trigonelline, brusatol	Inhibits NRF2	[67]
Thioredoxin	Ferroptocide	Covalently inhibits thioredoxin, causing cells susceptible to lipid peroxidation	[110]

*ACSL4* acyl-CoA synthetase long-chain family member 4, *BAP1* BRCA1-associated protein 1, *CoQ*_*10*_ ubiquinone, *CYB5R1* NADPH-cytochrome P450 reductase chrome b5 reductase, *GPX4* glutathione peroxidase 4, *GSH* glutathione, *HMG* 3-hydroxy-3-methylglutaryl, *HO-1* heme oxygenase 1, *IKE* imidazole ketone erastin, *IRP2* iron-regulatory protein 2, *LOXs* lipoxygenases, *LPCAT3* lysophosphatidylcholine acyltransferase 3, *NADPH* nicotinamide adenine dinucleotide phosphate, *NCOA4* nuclear receptor coactivator 4, *NOXs* NADPH oxidases, *NRF2* nuclear factor erythroid 2-related factor 2, *PE* piperazine erastin, *POR* NADPH-cytochrome P450 reductase, *ROS* reactive oxidant species, *RSL3* (1S, 3R-)RSL3, *SLC7A11* solute carrier family 7 member 11, *SLC39A14* solute carrier family 39 member 14, *SQLE* squalene epoxidase, *SQS* squalene synthase, *TFR1* transferrin receptor 1, *TXNRD1* thioredoxin reductase 1.

Many ferroptosis inducers do not fall into any of these four FIN classes and are summarized in Table 2. For example, the compound ferroptocide was recently identified as a potent ferroptosis agonist that covalently binds to thioredoxin (TXN), a 12-kDa ubiquitous oxidoreductase in the thioredoxin antioxidant system [110]. This binding is similar to the mechanism used by other ferroptosis inducers such as RSL3 and its analogs [103, 106, 107, 111]. The chloroacetamide-mediated activity of RSL3 and its analogs results in low target selectivity, as RSL3 can covalently modify a subset of non-GPX4 selenoproteins at the active site [106, 107, 112]. Similarly, the putative off-target effects of this compound may be attributed to its extensive covalent interactions with the proteome in ferroptocide-treated cells, which could be optimized by improving the selectivity for ferroptosis and proteins of interest.

**Table 2:** Genetic and pharmacologic inhibitors of ferroptosis.

Target	Effector/reagent	Proposed mechanism	Reference(s)
Genetic inhibitors			
SLC7A11	NRF2	Activates antioxidant genes; Upregulates *SLC7A11*	[127]
NRF2	KEAP1	Stabilizes and regulates NRF2	[127]
Lipid peroxides	GPX4	Eliminates phospholipid hydroperoxides	[17, 103]
Lipid peroxides	TXNRD1	Eliminates phospholipid hydroperoxides	[55]
Cystine	SLC7A11	Component of system X_c_^-^; Antiporter for cystine	[14, 17]
System Xc-	CD44v	Binds to SLC7A11 to stabilize system X_c_^-^	[128]
Actin dynamic	HSPB1	Regulates iron uptake and GPX4 abundance	[129]
Iron storage	FTH1	Subunit of intercellular iron-storage protein	[127, 130, 131]
Iron metabolism	STEAP3	Metalloreductase converting Fe^3+^ to Fe^2+^	[132]
Iron transport	FPN	Exports iron and reduces intercellular iron accumulation	[28, 133]
Iron transport	CISD1	Inhibits mitochondrial iron uptake and respiratory capacity	[34]
Iron metabolism genes	FANCD2	Regulates iron metabolism and lipid peroxidation	[132]
BH_4_ synthesis	GCH1	Involved in BH_4_ synthesis	[60, 61]
S-adenosyl homocysteine hydrolase	DJ-1	Determines the formation of the S-adenosyl homocysteine hydrolase tetramer and its enzymatic activity	[134]
CoQ_10_	FSP1	Converts CoQ_10_ to CoQ_10_H_2_, reducing membrane phospholipid peroxidation	[15, 16]
CoQ_10_	DHODH	Converts CoQ_10_ to CoQ_10_H_2_ in mitochondrial	[63]
PI3K-AKT-mTOR signaling pathway	mTORC1	Regulates amino acid metabolism and upregulates SREBP1/SCD1-mediated lipogenesis	[23]
SHP1-NF-κB- LCN2	LIFR	Suppresses NF-κB signaling through SHP1, leading to downregulation of LCN2	[99]
Pharmacologic inhibitors			
Iron chelators	Deferoxamine, deferoprone, deferasirox, cyclipirox	Chelate iron and block iron-dependent lipid peroxidation	[17, 135]
Lipophilic antioxidation	Fer-1, Lip-1, UAMC-3203, TEMPO, MitoTEMPO, α-tocopherol, trolox, BTH, CoQ_10_, idebenone, BH_4_, bazedoxifene, 2-(1-(4-(4-methylpiperazin-1-yl)phenyl)ethyl)-10*H*-phenothiazine	Inhibit lipid peroxidation	[4, 15–17, 23, 61, 115, 136]
LOX inhibitors	Zileuton, AA-861, PD-146176	Suppress LOXs and blocks LOX-induced lipid peroxidation	[111, 113, 137]
Amino acid metabolism	Glutathione, *N*-acetylcysteine, β-mercaptoethanol	Recover intracellular cysteine	[17]
Lipid peroxidation	Deuterated polyunsaturated fatty acids (D-PUFAs)	Suppress the initiation and propagation of lipid peroxidation	[138]
Protein synthesis	Cycloheximide	Blocks ferroptosis induced by system X_c_^-^	[17]
GPX4	PKUMDL-LC-101-D04	Activates GPX4 by allosteric activity	[117]
ACSL4	Rosiglitazone, pioglitazone, troglitazone	Inhibits ACSL4	[41]
DPP4	Vildagliptin, linagaliptin, alogliptin	Suppress DPP4-dependent lipid peroxidation	[67]
Selenoproteins	Selenium, methylselenocysteine, selenocystamine	Replenishes of GPX4 and other selenoproteins	[56, 139, 140]
N/A	Necrostatin-1, necrostatin-1f	Suppress ferroptosis in a RIPK1-independent manner	[4, 114]

*ACSL4* acyl-CoA synthetase long-chain family member 4, *AKT* AKT serine/threonine kinase, *BTH* butylated hydroxytoluene, *BH*_*4*_ tetrahydrobiopterin, *CD44v* CD44 variant, *CISD1* CDGSH iron-sulfur domain‒containing protein 1, *CoQ*_*10*_ ubiquinone, *CoQ*_*10*_*H*_*2*_ ubiquinol, *DHODH* dihydroorotate dehydrogenase, *DPP4* dipeptidyl peptidase-4, *FANCD2* Fanconi anemia complementation group d2, *Fer-1* ferrostatin-1, *FPN* ferroportin, *FTH1* ferritin heavy chain 1, *FSP1* ferroptosis suppressor protein 1, *GCH1* guanosine triphosphate cyclohydrolase 1, *GPX4* glutathione peroxidase 4, *HSPB1* heat shock protein b1, *KEAP1* kelch like ECH associated protein 1, *LCN2* lipocalin 2, *LIFR* leukemia inhibitory factor receptor, *Lip-1* liproxastatin-1, *LOX* lipoxygenases, *mTORC1* mechanistic target of rapamycin complex 1, *NRF2* nuclear factor erythroid 2-related factor 2, *PI3K* phosphoinositide-3-kinase, *RIPK1* receptor interacting protein kinase 1, *SCD1* stearoyl-CoA desaturase, *SHP1* SH2-containing protein tyrosine phosphatase, *SLC7A11* solute carrier family 7 member 11, *SREBP1* sterol regulatory element-binding protein 1, *STEAP3* six-transmembrane epithelial antigen of prostate 3, *TXNRD1* thioredoxin reductase 1, *N/A* not applicable.

#### Ferroptosis inhibitors

Ferroptosis can be inhibited using three main approaches (Table 2): iron chelation, preventing lipid peroxidation, and scavenging lipid peroxides. Of these three strategies, iron chelators and lipophilic antioxidants are widely accepted ferroptosis inhibitors. Iron chelators such as deferoxamine (DFO), deferiprone, and ciclopirox chelate iron and prevent the propagation of lipid peroxidation by limiting the Fenton reaction [17]. To date, a small handful of iron chelators have been approved by the FDA or have undergone clinical trials to treat iron overload-related diseases, transplantation, organ injury, and HCC. Lipophilic antioxidants, including α-tocopherol, Fer-1, and Lip-1, function as radical scavengers to reduce lipid peroxides and are effective at blocking ferroptosis [4, 17]. In mouse models of diseases such as ALI, liver fibrosis, NAFLD, and NASH, treatment with lipophilic antioxidants was shown to prevent disease progression and extend life expectancy. However, several limitations may preclude the use of Fer-1 and Lip-1 in clinical settings, including their possible unsatisfactory pharmacokinetics and pharmacodynamics profiles. For example, the biological half-life of Fer-1 is only a few minutes, which is generally unacceptable for clinical applications. With respect to the long-term use of ferroptosis inhibitors in patients with chronic conditions, previously undefined side effects may also appear in these patients. Notably, ACSL4 inhibitors such as rosiglitazone, troglitazone, and pioglitazone were previously reported to inhibit ferroptosis in a lipoxygenase-dependent manner; however, this finding was challenged by the finding that these ACSL4 inhibitors have inherent antioxidant activity [113].

Importantly, necrostatin-1 (Nec-1), a widely used inhibitor of necroptosis, has been suggested to inhibit both necroptosis and ferroptosis. For example, Nec-1f, a highly selective inhibitor of RIPK1 (receptor interacting protein kinase 1) has been shown to simultaneously inhibit necroptosis and ferroptosis in primary kidney tubules and mouse cardiac transplantation models [114]. Future studies are warranted to identify the mechanism underlying this dual inhibition as well as potential targets for treating pathological conditions associated with both necroptosis and ferroptosis. It is also interesting to note that Nec-1 has a better pharmacokinetics profile than Fer-1, and the maximum concentration of Nec-1f in tissues is relatively well tolerated [114–116], suggesting that Nec-1f may have better translational potential than Fer-1.

An number of ferroptosis-inhibiting compounds have been identified, including—but not limited to—beta-mercaptoethanol, selenium, cycloheximide, dopamine, and glutaminolysis inhibitors (Table 2). To the best of our knowledge, however, no clinical trials have been performed to test ferroptosis inhibitors in liver disease [3]. Given our limited understanding of ferroptosis-related pathways and regulatory mechanisms in a wide range of disease conditions, many questions remain. Regardless, ferroptosis inhibitors have been shown to be efficacious, at least in preclinical studies. Thus, future studies should focus on developing more potent ferroptosis inhibitors with improved drug properties, ideally leading to clinical testing.

### Future directions and perspectives

**Fig. 5:**
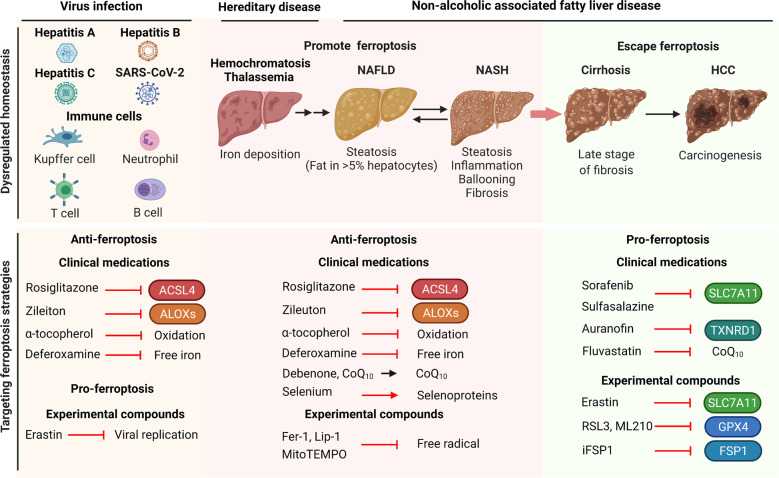
Strategies for targeting ferroptosis in treating liver disease. Dysregulated iron metabolism has been implicated in the development of a variety of hepatic diseases, including viral infectious liver disease, iron-overload disease, and the progression from NAFLD to HCC. These liver diseases can be treated using either ferroptosis inhibitors (middle panel) or ferroptosis inducers (right panel). Infectious liver diseases can be treated using ferroptosis inhibitors and ferroptosis inducers for host cells and viruses respectively (left panel). GPX4, glutathione peroxidase 4; ACSL4, acyl-CoA synthetase long-chain family member 4; ALOXs, arachidonate lipoxygenases; CoQ_10_, ubiquinone; Fer-1, ferrostatin-1; FSP1, ferroptosis suppressor protein 1; HCC, hepatocellular carcinoma; Lip-1, liproxstatin-1; NAFLD, non-alcoholic fatty liver disease; NASH, non-alcoholic steatohepatitis; SLC7A11, solute carrier family 7 member 11; TXNRD1, thioredoxin reductase 1.

Given the emerging role of ferroptosis in the pathogenesis of various liver diseases, ferroptosis serves as a promising therapeutic target (Fig. 5). Further studies are warranted in order to determine the precise role of ferroptosis at the cellular, tissue, and systemic levels. In the context of liver disease, more evidence is needed in order to provide a complete view of the dynamic processes by which ferroptosis drives the initiation of inflammation leading to liver fibrosis, cirrhosis, and—ultimately—carcinogenesis.

Based on well-established ferroptosis targets, significant effort has gone into drug design, such as designing allosteric activators of GPX4 [117]. Screening existing compound libraries and FDA-approved drugs will also likely help identify novel ferroptosis modulators and their underlying mechanisms. In this respect, high-throughput functional screening, automation, and artificial intelligence-based approaches may accelerate the development of ferroptosis-targeted drugs. Moreover, understanding the structure-function relationship and optimizing small molecules may help accelerate the development of new drugs. Indeed, an optimized Fer-1 molecule with an improved ADME (absorption, distribution, metabolism, and excretion) profile is expected to move into preclinic and clinic trials [115]. In addition, pharmaceutical studies are needed in order to successfully deliver the drugs to specific organs such as the liver with maximum safety and efficacy in a wide range of disease settings.

Although much progress has been made in our understanding of the pathological roles of ferroptosis in liver diseases, several critical questions remain to be addressed for further clinical development of ferroptosis-targeted therapies. First, what physiological role, if any, does ferroptosis play in the liver? Second, can we identify reliable, sensitive biomarkers of ferroptosis in liver disease? Third, when should we target ferroptosis in specific pathological liver conditions and/or disease stages? Fourth, in treating liver cancer, can we activate ferroptosis specifically in cancer cells without affecting healthy cells? Tackling the key scientific issues discussed in this review will improve our understanding of the precise role that ferroptosis plays in various pathophysiological liver conditions, thus providing a new scientific rationale for targeting ferroptosis in order to prevent and treat liver disease.
